# Adult tobacco use practice and its correlates in eastern Ethiopia: A cross-sectional study

**DOI:** 10.1186/1477-7517-10-28

**Published:** 2013-10-31

**Authors:** Ayalu Alex Reda, Daniel Kotz, Sibhatu Biadgilign

**Affiliations:** 1 Population Studies and Training Center, Brown University, Providence, RI, USA; 2 Department of Public Health, College of Health Sciences, Haramaya University, P. O. Box 138 Harar, Ethiopia; 3 CAPHRI School for Public Health and Primary Care, Maastricht University Medical Centre, Maastricht, the Netherlands; 4 Department of Epidemiology and Biostatistics, College of Public Health and Medical Sciences, Jimma University, Jimma, Ethiopia

**Keywords:** Smoking, Tobacco, Cigarettes, Ethiopia, Practice, Attitude, Rural, Population, Correlates

## Abstract

**Background:**

There is paucity of data on the smoking habits of rural populations in developing countries. This study aimed to explore cigarette smoking practices of a rural community in Ethiopia.

**Methods:**

A community based cross-sectional study was conducted among 548 individuals from a random sample of households in a rural town and its surrounding rural districts. Descriptive statistics and logistic regression were performed.

**Results:**

Twenty-eight percent (95% CI: 24.3% - 31.6%) of the respondents were current smokers. A total of 105 (68%) smokers expressed an interest to quit while 37 (34%) had tried to quit previously but without success. There was high exposure to second-hand smoke: 285 (52%) homes allowed indoor smoking, and in 181 (33%) indoor smoking took place daily. Current smoking was strongly associated with male sex (OR = 83.0; 95% CI: 11.5 – 599.0), and being a student was found to be protective of smoking (OR = 0.04; 95% CI: 0.005 – 0.05).

**Conclusion:**

Cigarette smoking is prevalent among the male rural town population in Ethiopia. In addition, a high level of exposure to indoor second-hand smoke exists. There is a need for investment in rural tobacco control, including educational campaigns and cost-effective smoking cessation services.

## Introduction

Tobacco use is one of the major preventable causes of premature death and disease in the world [1]. Tobacco use is increasing worldwide because of increased consumption in low-income countries [2-4]. As a result, a disproportionate share of the global tobacco burden falls on developing countries, where 84% of 1.3 billion current smokers reside [1]. The World Health Organization (WHO) attributes approximately 5 million deaths a year to tobacco. This number is expected to exceed 10 million deaths by 2020, with approximately 70% of these deaths occurring in developing countries [5]. Tobacco related deaths currently rank 2nd and 7th in middle-income and low-income countries respectively [6]. In Sub-Saharan Africa smoking caused just 100,000 deaths in 1990 and is projected to lead to deaths of more than 700,000 people in 2015 [7].

The tobacco epidemic is preventable. It was on this basis that World Health Assembly of the WHO unanimously adopted the Framework Convention on Tobacco Control (FCTC) in May 2003 [8]. This convention aims to tackle the epidemic at both national and global levels through a range of policy interventions. These interventions involve, among others, monitoring of tobacco use prevalence; offer of help for quitting tobacco use; warnings about the dangers of tobacco use; and bans on tobacco promotion and sponsorship [3,9].

National and international monitoring of tobacco use is considered essential for success in the fight against the tobacco epidemic [3]. In Ethiopia, nationally representative data on smoking prevalence and the effects of smoking are non-existent [10]. An earlier study reported an adult prevalence of 15.8% in 2005 [10], while a World Health Organization (WHO) estimate indicates a national tobacco use prevalence of 7.6% [3]. Most of these studies on smoking in the country have been conducted in urban populations or specific groups such as students [11,12] while rural areas and towns, where the majority (84.0%) of the population lives [13] are neglected in tobacco research [14]. So, the aim of this study was to assess cigarette smoking practice and its correlates among a rural town population in eastern Ethiopia.

## Methods

### Study area and period

A community based cross sectional study was conducted in Kersa town, eastern Ethiopia from February to July 2010. The source population for this study were individuals above the age of 15 years enrolled under the Kersa Demographic Surveillance and Health Research Center (KDS -HRC). The KDS-HRC is located in Kersa district, Oromia region, Ethiopia. It is a demographic and mortality surveillance site intended to monitor mortality and morbidity in the study area. The study site has twelve Kebeles (the smallest administrative unit in Ethiopia) with 10,256 households and a population of 48,192 [15]. Our sample was taken from the rural town of Kersa and the surrounding rural districts which had 3,004 households representing 11,072 persons at the time of the study.

### Data collection procedure

In 1998, the WHO, US Centers for Disease Control and Prevention (CDC), and the Canadian Public Health Association (CPHA) developed the Global Tobacco Surveillance System (GTSS) to assist countries in establishing tobacco use control and prevention programs [5]. One of the survey tools developed by the GTSS is the Global Adult Tobacco Survey (GATS) questionnaire which is aimed to assess smoking among adults. It contains items on smoking and its magnitude, knowledge of the risks of smoking, smoking cessation etc. The GATS questionnaire [16] was translated into Oromifa (the local language) and adopted for data collection through interviews.

In order to avoid sampling of more than one person in the same household and increase representation, we selected households first and then the individual. Sample size was calculated using a single proportion [17] sample size formula with the following assumption: 95% confidence interval (CI), prevalence of smoking of 25%, margin of error of 2.8%, and a 10% non-response rate. This gave a sample of 600 individuals. Six hundred households were then randomly sampled using the KDS-HRC digital database as a sampling frame. Data collectors visited the selected households and enumerated members above the age of 15 years. Then one person was selected for interview from this list using the random number generator table included in the standardized GATS questionnaire. The investigators and two site personnel supervised the data collection process. The questionnaire was pretested among 38 individuals taken from a similar population in a nearby district.

### Data analysis

Data were entered and analyzed using IBM® SPSS® Statistics, version 15 for Windows. Descriptive statistics and logistic regression were performed. These included binary as well as multiple logistic regression models where status of smoking was entered as the dependent variable and socio-demographic measurements as independent variable. Proportions and their 95% confidence intervals were calculated to assess cigarette smoking practice. P-values less than 0.05 were considered significant.

### Operational definitions

The responses to the questionnaire item “Do you currently smoke tobacco?" were used for assessing current smoking, and "Have you smoked tobacco daily in the past?" for former smoking.

### Ethical clearance

Ethical clearance was obtained from the institutional research ethics review (IRB) board of Haramaya University. Verbal informed consent was obtained from respondents.

## Results

Five hundred forty-eight of the 600 respondents responded to interviews, providing a response rate of 91.3%. There were 405 male respondents (75.1%) and the mean (SD) age of the respondents was 35.0 (15.0) years (Table 1).

**Table 1:** Background and socio-economic characteristics of the sampled population (N = 548)

**Characteristics**	**Male**	**Female**	**Total**
Sex	405 (75.1)	134 (24.9)	539
Age, mean (SD)	35.0 (13.6)	34.0 (18.4)	35.0 (15.0)
Religion			
Christian	71 (17.7)	42 (30.9)	115 (21.3)
Muslim	328 (81.8)	92 (69.2)	424 (78.3)
Others	2 (0.5)	0 (0.0)	2 (0.4)
Education			
Illiterate	162 (40.1)	78 (58.2)	243 (44.7)
Below secondary school	168 (41.6)	30 (22.4)	200 (36.8)
Secondary school completed	30 (7.4)	14 (10.4)	45 (8.3)
High school completed	23 (5.7)	7 (5.2)	30 (5.5)
College completed	16 (4.0)	4 (3.0)	20 (3.7)
Postgraduate	4 (1.0)	0 (0.0)	4 (0.7)
Marital status			
Single	93 (22.4)	102 (77.2)	198 (28.6)
Married	309 (76.3)	30 (22.4)	343 (63.4)
Work status			
Governmental employee	55 (13.6)	11 (8.3)	70 (12.9)
Non-governmental employee	8 (2.0)	2 (1.5)	10 (1.8)
Self-employed	279 (69.2)	38 (28.8)	319 (58.9)
Student	46 (11.4)	27 (20.5)	73 (13.5)
Homemaker	3 (0.7)	24 (18.2)	27 (5.0)
Unemployed	5 (1.2)	7 (5.3)	12 (2.2)
Other	7 (1.7)	23 (17.4)	31 (5.7)

Figures are presented as N (%), unless otherwise stated.

### Practice and attitudes toward smoking

One hundred fifty-one (28%) respondents reported to smoke daily, whereas 6 (1.1%) smoked on a non-daily basis (1.1%). The proportion of current smokers (daily and non-daily smoking at the time of the study) was 38.6% among males and 0.8% among females. Twenty-two (4%) of the respondents were former smokers, from which 10 (1.8%) were daily smokers. The mean (SD) age of smoking initiation was 21.1 (6.4) years. Twenty-six (5%) of the respondents used smokeless tobacco, mainly in the form of chewing. From these 9 (1.7%) used it daily. The mean (SD) number of cigarettes smoked per week by smokers was 47.2 (51.4), showing a significant variation from 1 to 168 cigarettes. This amounts to an average of 6.7 cigarettes smoked per day (range 24). The mean (SD) expense of the last cigarette purchase was 4.7 (6.1) birr (currency of Ethiopia) which amounts to $0.9 ($1.1) in 2010 purchasing power adjusted dollars.

Thirteen (8.7%), 21 (14.0%), 24 (16.0%), and 92 (61.3%) smokers reported to smoke the first cigarette within respectively 5, 6–30, 31–60, and more than 60 minutes after waking up in the morning. About 34.0% (49) of smokers had tried to stop smoking in the past. The mean (SD) number of days of cessation was 5.9 (13.5). A total of nineteen (27.9%) smokers who visited a health facility in the past year were asked by health personnel about the use of tobacco and 16 (24.6%) were advised to quit. The main method used for smoking cessation was counselling (25%). Regarding plans to quit smoking, 16 (10.3%), 31 (20.0%), 58 (37.4%) respondents aimed to quit respectively within 6 months, 12 months, and some day but not within 12 months. Thirty-seven (23.9%) smokers were not interested in quitting. Plan to quit was negatively associated with number of cigarettes smoked per week (*X*^*2*^ = 34.3, df = 3, p < 0.001).

### Factors associated with smoking

In the multivariate analysis using logistic regression current smoking was strongly associated with male sex (OR = 83.0; 95% CI: 11.5–599) and students had lower odds of smoking than those of employed respondents (OR = 0.04; 95% CI: 0.005–0.05). Age was statistically significant in the bivariate analysis (OR = 1.02; 95% CI: 1.01-1.03) but not after adjustment for other variables (Table 2).

**Table 2:** Predictors of currents smoking in Kersa town, eastern Ethiopia

**Predictors**	**Crude OR (95% CI)**	**Adjusted OR (95% CI)**^**¥**^
Age	1.02 (1.01 – 1.03)	1.01 (0.99 - 1.03)
Religion		
Muslim	3.3 (1.8 – 5.9)	1.9 (0.8 – 4.6)
Christian	1.0	1.0
Sex		
Male	83.0 (11.5 – 599)	87.6 (10.8 – 708.6)
Female	1.0	1.0
Education		
Illiterate	3.9 (2.8 – 5.4)	2.3 (0.9 – 6.2)
Below secondary school	2.0 (1.4 – 2.8)	1.4 (0.6 – 3.5)
Higher	1.0	1.0
Marital status		
Married	4.0 (2.5 – 6.3)	1.5 (0.8 – 2.9)
Single	1.0	1.0
Work status		
Employed	1.0	
Self-employed	3.5 (2.5 – 4.8)	1.5 (0.6 – 4.1)
Student	0.03 (0.01 – 0.07)	0.04 (0.005 – 0.05)
Unemployed	1.5 (0.7 – 3.1)	0.23 (0.025 – 2.26)
Others	0.08 (0.03 – 0.22)	1.5 (0.2 – 11.1)

^¥^Negelekerke R^2^ = 40.3%; Cox & Snell R^2^ = 28.2%; -2LL = 446.7; Hosmer and Lameshaw test p 0.234; Correct classification 75.1%.

### Indoor smoking

Two hundred ten (40%) households allowed indoor smoking and sixty-seven (12.2%) allowed it with exceptions. One hundred sixty-six (31%) homes never allowed indoor smoking while ninety two (16.9%) did not have rules on second hand smoke. In one-third of homes (33%) smoking took place daily. Four hundred (73.0%) respondents believed that second hand smoking causes health problems. With respect to the health consequences of second hand smoke, heart disease was reported by 81.9% (357) of the respondents; respiratory problems by 89.7% (401); and lung cancer by 84.7% (371).

## Discussion

Our findings indicate that smoking is practiced highly (28.6%) among the population studied. Being male was a strong predictor of tobacco use. Smokers consumed an average of 6.7 (range 24) cigarettes, where the last purchase cost them an average of 0.9 (SD = 1.1) dollars. About 68% of smokers had interest to quit while 34% had tried to quit previously but without success. There was a high exposure to second-hand smoke; 52% of homes allowed indoor smoking and in 33% smoking took place daily.

Compared to findings from other parts of the country [11,12,18], the practice of smoking in this rural population is very high. In this study, the prevalence of smoking was 0.2% among females. Our findings of smoking practice are also more than the 8.1% and 13.3% rates of smoking reported among university students and instructors respectively in Northern Ethiopia [11,12]. In fact, the cigarette smoking prevalence in the current study population is much higher than the national average of 7.2% [3]. Interestingly, it is even higher or comparable to reports from countries such as Kenya 22.9% [19], Tanzania 21% [19], and Tunisia 30.4% [20], where smoking practices are considered higher by African standards [19].

In a similar manner to other reports in Ethiopia and elsewhere, males contribute most to the habit of smoking [11,12,18,20,21]. The 2005 Ethiopian Demographic and Health Survey (EDHS) estimated the prevalence of smoking among males aged 15–49 years at 9%, where as for females it was 2% [22]. A survey of a rural town population in southern Ethiopia by Schoenmaker and colleagues reported that 5.8% of the population and 15.4% of males smoked [18]. In reports from Africa [14,20,23,24] and elsewhere [2,14,21], smoking was independently associated with male sex after adjustment for other variables such as age and occupation. This could be because of the conservative culture prevalent in the study area where females are not allowed to use tobacco and alcohol. In this study, students had lower levels of smoking compared to the rest of the sample. Some studies report higher smoking rates among the unemployed or little educated [14,21,25], others higher education groups [18,25]. Compared to other faiths, being a Muslim is commonly reported as a predictor of smoking in studies conducted in other parts of Ethiopia [11,18] and outside [14]. In this study, while it was significant on bivariate analysis its effect diminished on multivariate analysis (p = 0.11).

Even though differences in definitions of tobacco use may contribute to differences between studies, their contribution to very high differences is limited. The primary factor that could explain the high level of tobacco use in this rural population is the fact that khat (*Catha edulis*), a stimulant leaf composed of the psychoactive chemical methamphetamine, is highly consumed along with tobacco and is extensively farmed [11,18]. Khat is consumed in private and at social events by all age groups and both sexes since it is part of the culture of the present population. In the khat chewing ritual, smoking helps to heighten the stimulant effect of khat and to break the negative psychological states such as depression that ensue when the stimulation from the khat subsides.

Not only active smoking but also second-hand smoking is expected to have a high impact on health. In this study population a large percentage of homes (52%) allowed indoor smoking. This figure is higher than a report from Georgia in the US where only 12% of homes allowed smoking [26] but lower than the 75% report from the Dominican Republic [2]. This is a risky situation as rural homes in Ethiopia as elsewhere also grapple with indoor smoke arising from burning biomass fuel [27]. It is reported that children particularly face allergic and respiratory problems as a result of exposure to second hand smoke [28] and is a cause of the under-recognized problem of Chronic Obstructive Pulmonary Disease (COPD) among adults in the developing world. Interestingly, smokers knew more about the impacts of second hand smoke than non-smokers. This may explain the high rate of lenience by households toward smoking at home in this study as non-smokers may underestimate the risks involved and fail to take prohibitive action. For instance, a study in the US indicated that among respondents who are smokers and live with children, 85% reported that smoking occurs regularly in their homes [29].

Smokers in this study seem to smoke fewer cigarettes per day (mean = 6.7) in comparison to reports from other countries [18,20,30] such as Tunisia where a mean consumption of 17.7 cigarettes per day was reported [20]. A different report from a rural town in southern Ethiopia indicated a similar cigarette consumption pattern to this study, where respondents smoked a mean of 6.6 cigarettes per day [18]. Reports among students and university instructors in Northern Ethiopia indicated a consumption rate of 3.2 and 8.6 cigarettes per day respectively. It is also acknowledged that dissimilarity in the definition of smoking/tobacco use may explain the apparent difference in consumption patterns and quit attempts.

The smokers in this study spent an average of 0.9 dollars on cigarettes in their last purchase. This is a large sum in a country where nearly one third of the population lives below the poverty line (an income of ≤ $1.25 per day) [31]. It is reported that low socio-economic tobacco users tend to use more and spend a significant share of their income on tobacco compared to wealthy individuals [2,3,14,21,32]. This leads not only to diversion of personal and household income [32], but also to a greater level of suffering for both users and their families as second-hand smokers [28]. This becomes a serious burden to poor countries as they have to also grapple with the consequences of tobacco use on top of their traditional infectious disease challenge [4,6,33]. Smoking may ultimately lead to death of the bread winner and cut off livelihood of the family since in rural and poor families ability for mobility and physical work is the main form of income [33].

A positive finding of this study is the high interest shown by smokers towards quitting. About 68% of smokers reported an interest to quit. This is higher than the 57% proportion reported from a nearby city, Dire Dawa, Ethiopia [34]. For instance, a study from Tunisia indicated that only 9.4% of the smokers had made a quit attempt in comparison to 34% in this study and a 59.9% reported from a nearby city [34]. Only 24.5% of the smokers that went to a health facility were advised to quit smoking. Given the high level of interest to quit by respondents, we believe the opportunity of smoking cessation interventions in the health facilities is underutilized. However, even when assuming a willingness to assist smokers to quit, it is our view that the necessary skills and medications are unavailable.

This study could be a good addition to the literature on smoking in resource poor and rural settings. The strengths of this study are the use of the Global Adult Tobacco Survey (GATS) questionnaire, a standardized international questionnaire with carefully chosen questions to assess tobacco use and also enable comparison of data across settings; and use of representative sample drawn from a sampling frame. The potential limitation of the study is the possibility of response bias especially among female respondents. Given the conservative cultural environment of the country, they may be less encouraged to reveal their substance abuse habits. This is more so when they are requested for such information through face-to-face interviews. However, given the low literacy level in the study population, self-administered questionnaires are not feasible and we were left with interviews as the only means of data collection.

In conclusion, there was a high practice of consumption of cigarettes among males in the study population. The prevalence of smoking among females was very minimal. There was a high level of indoor air pollution from second hand smoke. The majority of smokers were interested in quitting smoking. Concerted effort is required from community leaders, health workers, and policy makers to avert the existing tobacco use and its consequences. Specifically, there is a need for investment in tobacco control, including educational campaigns and development of cost-effective smoking cessation services such as counselling in smokers presenting at health care institutions in the study area.

## Competing interests

All authors declare that they have no conflict of interest associated with the publication of this manuscript.

## Authors’ contributions

AAR conceived and designed the study and collected data in the field, performed analysis, wrote a draft of the manuscript. DK assisted with the design, interpretation of data and the critical review of the manuscript. SB performed interpretation of data, and critically reviewed the manuscript. All authors approved and read the final manuscript. All authors participated in critical appraisal and revision of the manuscript.
